# The effect of inhibition of the Na^+^/H^+^ exchanger on the development of hypertrophy in hypertrophic cardiomyopathy

**DOI:** 10.1186/1532-429X-13-S1-P290

**Published:** 2011-02-02

**Authors:** Wessel P Brouwer, Arjan C Houweling, Marcel G Nederhoff, Tjeerd Germans, Marcel M van Borren, Gerard Pasterkamp, Jolanda van der Velden, Albert C van Rossum

**Affiliations:** 1 VU University Medical Center, Amsterdam, Netherlands; 2 University Medical Center, Utrecht, Netherlands; 3 Jeroen Bosch Ziekenhuis, 's Hertogenbosch, Netherlands

## Introduction

In hypertrophic cardiomyopathy (HCM), the pathogenesis of asymmetrical left ventricular (LV) wall thickening is based upon a complex interplay of molecular pathways and is still largely unclarified. Since cariporide (an Na^+^/H^+^ exchange blocker) has been proven effective in several pressure overload (animal) models by inhibiting the hypertrophic response, we hypothesized that cariporide might prevent or diminish the development of hypertrophy seen in HCM. Therefore, we treated transgenic mice (homozygous cMyBP-C null mice) with cariporide and subjected them to cardiomagnetic resonance imaging (CMR).

## Purpose

To determine the effect of cariporide on the development of LV hypertrophy in hypertrophic cardiomyopathy.

## Methods

We used 19 male homozygous cMyBP-C null mice in our study, of which 10 animals were treated with standard animal chow containing 6000 ppm cariporide, beginning at 5 weeks of age for 2 months. In order to evaluate LV mass and function, the animals underwent state-of-the-art CMR-imaging at 5 weeks and at 3 months of age. The control group consisted of wildtype animals (n=14) who were scanned at 6 months of age. A state-of-the-art 9.4 T scanner (Bruker, Erlangen) was used for CMR acquisitions. Off-line analysis was performed using dedicated software (Qmass, Medis, Leiden).

## Results

There were no significant differences in global and regional LV mass and LV function between the treated and untreated mice at 5 weeks (data not shown) and 3 months of age (depicted in table 1). Furthermore, transgenic mice of 5 weeks old already showed a significant increased LV mass (corrected for bodyweight) with respect to wildtype animals (3.37 ± 0.43 (x10^-3^) vs 2.07 ± 0.31 (x10^-3^), p<0.01).

**Table 1:** CMR-derived LV dimensions and mass of homozygous cMyBP-C null mice at 3 months of age.

	Cariporide (n=10)	Non-treated (n=9)	P-value
LV mass (mg)	94.6 ± 12.6	88.6 ± 8.3	ns
LV mass/body weight (10^-3)^	3.16 ± 0.34	3.07 ± 0.43	ns
LVEDV (ul)	91.8 ± 13.4	89.7 ± 12.7	ns
LVESV (ul)	68.7 ± 11.3	65.8 ± 12.9	ns
LVEF (%)	25.4 ± 3.9	27.0 ± 4.1	ns
Mean septal wall thickness (mm)	0.82 ± 0.06	0.80 ± 0.62	ns
Mean LV wall thickness (mm)	0.89 ± 0.09	0.84 ± 0.08	ns
Maximal septal wall thickness (mm)	1.17 ±0.17	1.15 ± 0.14	ns
Maximal LV wall thickness (mm)	1.29 ± 0.18	1.32 ± 0.16	ns

## Conclusions

Our study demonstrated that cariporide has no effect on the regression of LV hypertrophy in homozygous cMyBP-C null (HCM) mice after two months of treatment. Apparently, blockade of the Na^+^/H^+^ exchanger was unable to reverse established hypertrophy in HCM. Whether cariporide is able to prevent hypertrophy in HCM needs further evaluation.

